# Therapeutic Decisions and Implications in Patients With Attention Deficit Hyperactivity Disorder Versus Bipolar Disorder: Key Insights for Clinicians

**DOI:** 10.7759/cureus.67588

**Published:** 2024-08-23

**Authors:** Ngozi Adaralegbe, Ayotomide Oyelakin, Omobusayo Omotayo

**Affiliations:** 1 Department of Psychiatry and Behavioral Sciences, University of Texas Health Science Center at Houston - McGovern Medical School, Houston, USA; 2 Department of Medicine, Nova Southeastern University Dr. Kiran C. Patel College of Osteopathic Medicine, Fort Lauderdale, USA

**Keywords:** hyperactivity, impulsivity, bipolar disorder, pediatric bipolar disorder, adhd

## Abstract

Attention deficit hyperactivity disorder (ADHD) and pediatric bipolar disorder (BD) are conditions that often manifest in childhood and can persist into adulthood. Due to the similarities in their clinical presentations, it is crucial for clinicians to have a thorough understanding of these disorders. Accurate differentiation of symptoms is essential for making precise diagnoses, as this directly influences treatment decisions and affects the overall functioning and quality of life of those impacted.

Considered here is the case of a teenage male who initially presented with impulsivity and was diagnosed with ADHD. However, upon further evaluation, his diagnosis was revised to BD. This case highlights the importance of diagnostic accuracy in clinical practice. Correctly identifying these conditions ensures timely and appropriate treatment, which can significantly alter the life trajectory of affected individuals. This encompasses improvements in health outcomes and better functioning in social, occupational, and other aspects of their lives when treatment is appropriately tailored.

## Introduction

Attention deficit hyperactivity disorder (ADHD) and pediatric bipolar disorder (BD) typically emerge in children and adolescents, often persisting into adulthood. It's crucial for clinicians to have a solid understanding of these disorders to prevent underdiagnosis, overdiagnosis, or misdiagnosis. When a patient presents with symptoms like impulsivity, hyperactivity, distractibility, rapid speech, and increased energy, clinicians consider various differential diagnoses, including ADHD and PBD. The prevalence of ADHD has been steadily increasing over the past decade. The National Health Interview Survey (NHIS) reported a 33% rise in prevalence between 1997 and 2008, while the National Survey of Children’s Health (NSCH) indicated a 42% increase between 2003 and 2011 [1]. The co-occurrence of PBD in children and adolescents with ADHD is documented in the literature, with rates as high as 5.86%, and even higher in adults, at 15.44% [2].

The clinical presentation of ADHD can pose challenges in distinguishing it from other psychiatric disorders due to overlapping features, leading to delays in diagnosis, treatment, and potentially worsening outcomes. With the absence of biomarkers and laboratory parameters, clinicians often rely on their clinical expertise for early recognition and accurate diagnosis. Studies have demonstrated that early and appropriate treatment of ADHD can alter its trajectory and the manifestation of other psychiatric comorbidities [3]. Despite a substantial body of literature aimed at guiding clinicians in recognizing these distinct disorders and developing treatment plans, community clinicians still face difficulties in identifying and diagnosing these disorders early [4]. This article aims to provide a clinical framework to enhance the understanding and management of these two distinct, sometimes co-occurring disorders.

## Case presentation

We present the case of a 17-year-old Caucasian male who was previously diagnosed with ADHD at age 10 and followed by his pediatrician. He was referred for an initial psychiatric evaluation at our community clinic at the age of 16. On evaluation, he presented with seven out of nine inattentive symptoms and seven out of nine hyperactivity/impulsivity symptoms, demonstrating high levels of impulsivity. His history included destructive behavior, association with risky peers, dishonesty, theft, running away from home for two to five days, breaking curfew, and defiance of authority. He reported episodic mood swings characterized by irritability, changes in sleep patterns, increased energy, overconfidence, heightened impulsivity, and substance use every three months. He admitted to daily vaping of nicotine and cannabis, along with occasional alcohol use, leading to intoxication.

Despite being a 10th grader at a local high school, he faced numerous disciplinary actions due to behavioral issues and was caught with drug paraphernalia during one episode. He had also been arrested and placed in juvenile detention for theft, driving under the influence, and violating probation during one of his mood episodes. Previous school psychological evaluations, at age 13, noted emotional and behavioral symptoms interfering with functioning, including frequent anger episodes, irritability, impulsivity, low frustration tolerance, poor motivation, and oppositional behavior.

Noteworthy is his family history of BD in first- and second-degree relatives, and intrauterine exposure to cannabis. Pharmacological trials included risperidone, which exacerbated aggression; dextroamphetamine, 20 mg, which worsened mood swings; and guanfacine extended-release (ER) 4 mg and clonidine 0.3 mg, with limited effectiveness. Subsequently, he was psychiatrically hospitalized due to severe insomnia, engaging in high-risk behavior such as theft, and exhibiting severe agitation with physically aggressive behavior at school. His diagnosis was revised to bipolar I disorder with comorbid ADHD.

He was initiated on Abilify, currently titrated to 20 mg daily for mood stabilization; guanfacine ER 3 mg for ADHD; and trazodone 50 mg for sleep, with positive results. He is currently stable on these medications, with regular clinic follow-up.

## Discussion

Clinical presentations of ADHD and BD

According to the Diagnostic and Statistical Manual of Mental Disorders, 5th Edition, Text Revision (DSM-5-TR) [5], BD is an illness characterized by a constellation of symptoms, including changes in mood, sleep, activity level, cognitive function, and overall ability to function. ADHD, on the other hand, is described as a neurodevelopmental disorder marked by impairing levels of inattention, disorganization, and/or hyperactivity-impulsivity. This impairment in functioning is typically observed across multiple settings, such as school and home. Individuals with ADHD can present with any of the following: combined type, predominantly inattentive type, or predominantly hyperactive-impulsive type, for a period of six months [5]. Research suggests that the combined subtype is associated with a higher prevalence of psychiatric comorbidities [6]. Approximately 65% of children diagnosed with ADHD are reported to meet diagnostic criteria for BD in adulthood [7].

Various factors contribute to the complex manifestations of ADHD and BD, making it challenging for clinicians to assess adequately. One such factor is the impaired ability of pediatric patients to self-report symptoms, coupled with a reliance on, or overdependence on, parents and school staff to retrospectively report symptoms. Substance use and some personality traits, like antisocial and borderline personality traits, are also prevalent among the adolescent population, and they can further complicate the clinical picture, posing significant challenges in navigating the presentations.

Diagnostic overlap

Inattentiveness, increased distractibility, and hyperactivity are typically indicative of ADHD, while BD presents with changes in neurovegetative functioning, mood swings, agitation, or aggressive behaviors, as evidenced in the case presented above. The challenge in delineating symptoms arises because the hyperactivity and behavioral dysregulation seen in ADHD can mimic the hypomanic or manic presentation in BD. An additional confounder is the presence of coexisting conduct symptoms or oppositional behavior. In the case described, there was a delay in diagnostic clarification and appropriate treatment, as some of the high-risk and dysregulated behavior was initially attributed to ADHD and impulse control issues. However, as the episodic nature of his increasing impulsivity became more apparent, the threshold for a BD diagnosis became more evident. 

Diagnostic differentiation of ADHD and BD

ADHD typically manifests in childhood, while the diagnosis of BD is more commonly made in adulthood. However, there are instances of BD being diagnosed in children and adolescents, albeit less frequently [8]. There exists a significant overlap in the diagnostic criteria of ADHD and BD, with both disorders featuring emotional lability, increased talkativeness, psychomotor agitation, and heightened distractibility. Some researchers have explored possible etiological relationships between these two disorders [9]. Family studies have consistently demonstrated an aggregation of ADHD and BD over the years, underscoring the importance of delineating between them [10]. The distinction between the two disorders is crucial, as it informs treatment strategies and long-term prognosis, which differ for each condition.

Most children with ADHD experience impairment in social and academic functioning by age 7, although symptoms may diminish in about 50% of cases as they transition into adulthood [11]. In contrast, individuals with BD typically present later than age 7 and exhibit a more episodic but persistent course due to the nature of the symptoms. This contrast in presentation - ADHD being chronic and non-episodic, and BD having an episodic nature - can aid in evaluating patients with challenging presentations. In the case described above, the episodic nature of the patient's impulsivity and engagement in high-risk behavior every few months was instrumental in clarifying the diagnosis.

**Figure 1:**
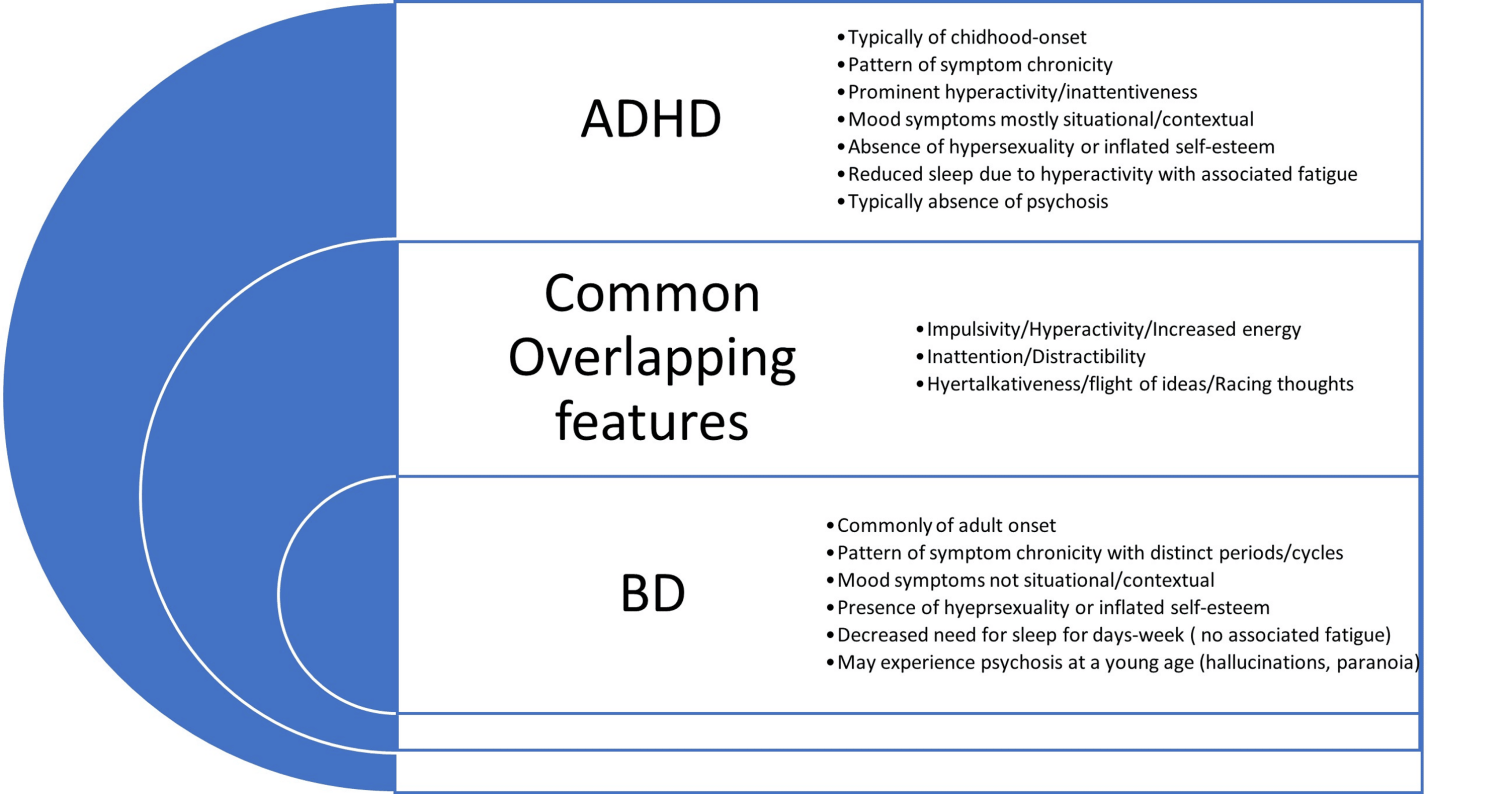
Overlapping features of ADHD and BD Image credit: Dr. Ngozi Adaralegbe ADHD: Attention deficit hyperactivity disorder; BD: Bipolar disorder

The manic episode of BD and ADHD presents a formidable challenge for clinicians. According to the DSM-5-TR criteria for ADHD [5], fundamental features include distractibility, over-activity, and hyper-talkativeness, which are also commonly experienced by individuals with BD during the manic phase (Figure 1), along with psychomotor agitation, inflated self-esteem, and decreased need for sleep.

Irritable mood has been reported in both ADHD and manic episodes of BD, even though not a prominent feature in ADHD, further complicating the diagnosis [12]. The impulsivity and inattention characteristic of ADHD can be mistaken for the manic or hypomanic episodes seen in BD, and vice versa. For example, "increased distractibility" could be confused with "inattentiveness" seen in ADHD inattentive type. Similarly, "increased activity" or psychomotor agitation seen in mania often resembles "hyperactivity" in ADHD. Furthermore, "talkativeness" in mania may resemble criteria of "talks excessively" or "blurts out an answer before a question has been completed" in ADHD. Irritability, a common symptom in both conditions, poses a diagnostic challenge, as it is also observed in various other mental health disorders such as generalized anxiety disorder, oppositional defiant disorder, major depressive disorder, and conduct disorder [12]. While ADHD typically presents with no mood disturbance, the co-occurrence of irritability in ADHD further complicates the diagnostic process.

Literature has evidence to elucidate the symptoms that best differentiate BD from ADHD. According to Geller et al., children with BD have predominantly manic symptoms such as grandiosity, flight of ideas, elation, hypersexuality, or decreased need for sleep [13]. Conversely, children with ADHD often experience worsening of their symptoms during a manic episode, with hyperactivity being the most prominent [13]. While children with BD may exhibit a reduced need for sleep during manic episodes, those with ADHD may display increased activity when getting ready for bed and eventually falling asleep.

To distinguish ADHD from BD, clinicians must identify the pressured speech with loose associations, flight of ideas, and grandiose delusions typically seen in mania, which are not typical characteristics of ADHD. Notably, children and adolescents with ADHD do not typically exhibit extreme thought disorders unless there are other comorbidities [14]. Furthermore, the increased energy seen in ADHD is typically not as extreme as that observed in mania, where individuals may go for multiple days without sleep. Another helpful practice in attempting to differentiate between BD and ADHD is to accurately ascertain the presence of the criterion “B” symptoms of mania [5], which are not usually present in ADHD.

Therapeutic decisions and implications

Recognizing whether ADHD is occurring in isolation or as a comorbidity with BD is pivotal in determining management decisions. The therapeutic implications should be carefully considered, especially when they co-exist. About 50% of children and adolescents diagnosed with ADHD and BD continue to use stimulants to target their ADHD symptoms, which has the potential to further dysregulate mood and trigger mania. A link between the use of stimulants and the development of BD symptoms has been postulated [15]. Despite numerous studies on ADHD and BD, there is limited data to guide the treatment of comorbid ADHD and BD. Existing literature recommends addressing BD first if it co-occurs with ADHD, ensuring mood stabilization before treating residual or persisting ADHD symptoms [16]. When a child or adolescent with ADHD is wrongly diagnosed with BD and commenced on a mood stabilizer, the mood stabilizer may help with some of the hyperactivity, impulsivity, and irritability, if present, but does not address inattention and cognitive/executive functioning symptomatology.

Existing literature is heterogeneous on the association between ADHD and BD, and clinicians must interpret this with caution. While some studies have shown increased rates of BD in individuals diagnosed with ADHD [17], other population-based studies have not demonstrated any association [18]. Few studies have identified that the earlier onset type of BD is associated with higher rates of ADHD diagnosis. Some studies have also shown an association between the current or prior use of stimulants in those diagnosed with ADHD and the early development of BD [19]. While some studies reveal a worrisome association between the use of stimulants and the early development of BD, or even substance use disorder, a study has also revealed no association between the use of methylphenidate and substance use disorder [20]. In situations where ADHD and BD are co-occurring, they are less likely than those with an isolated diagnosis of BD to effectively respond to mood-stabilizing treatment. About 92% of patients with ADHD and manic symptoms had remission of their symptoms after their stimulants were discontinued, while symptoms persisted in about 8% even after discontinuation of their stimulant medication [21]. Despite extensive documentation suggesting that stimulant use can potentially worsen mania or even psychotic symptoms, research indicates no worsening of manic or psychotic symptoms, with significant treatment response documented in children and adolescents with ADHD and comorbid BD on stimulants, especially in the early phase of treatment [21]. With that stated, mood stabilization should be the primary goal in individuals with comorbid ADHD and BD.

Early intervention and initiation of appropriate treatment positively impact the overall well-being of diagnosed individuals and their longer-term prognosis. While ADHD is typically managed with stimulants, such as methylphenidate and amphetamine-based compounds, as the first line, and non-stimulants, such as alpha-agonists, atomoxetine, and viloxazine, as the second line, BD is managed with mood-stabilizing and/or antipsychotic medication. Stimulants are notorious for inducing or precipitating manic or psychotic episodes; thus, great caution must be applied when using stimulants in patients with ADHD to avoid triggering manic or psychotic episodes in undiagnosed patients.

## Conclusions

The diagnostic accuracy of clinical conditions such as ADHD and pediatric BD is of paramount importance, as it directly informs timely and effective treatment strategies. Clinicians need to be well-versed in the wide range of clinical conditions that may present with similar symptoms, such as impulsivity. Bearing in mind the episodic nature of the symptoms, which are often non-situational, and specifically screening for other manic symptoms, such as grandiosity, could help differentiate these diagnoses. Manic patients display elevated energy and reduced need for sleep, while ADHD individuals are often exhausted after periods of hyperactivity. It is also important to know that both conditions can co-occur, often with severe symptoms and complications. Early intervention is key to mitigating the long-term effects of these disorders and can significantly enhance the quality of life for those affected.

Moreover, clinicians should be equipped to provide thorough guidance to all parties involved, including the patients and their families, to support them through the treatment process. This includes educating them about the nature of the disorder, the expected course of treatment, potential outcomes, and strategies for managing the condition in everyday life. Such holistic care is vital for fostering a supportive environment that promotes the best possible health outcomes and overall well-being for individuals living with these challenging disorders.
